# Repurposing Dimethyl Fumarate for Cardiovascular Diseases: Pharmacological Effects, Molecular Mechanisms, and Therapeutic Promise

**DOI:** 10.3390/ph15050497

**Published:** 2022-04-19

**Authors:** Shilu Deepa Thomas, Niraj Kumar Jha, Bassem Sadek, Shreesh Ojha

**Affiliations:** 1 Department of Pharmacology and Therapeutics, College of Medicine and Health Sciences, United Arab Emirates University, Al Ain P.O. Box 15551, United Arab Emirates; shilu.d.thomas@gmail.com; 2 Zayed Bin Sultan Center for Health Sciences, United Arab Emirates University, Al Ain P.O. Box 15551, United Arab Emirates; 3 Department of Biotechnology, School of Engineering and Technology (SET), Sharda University, Greater Noida 201310, India; niraj.jha@sharda.ac.in

**Keywords:** dimethyl fumarate, DMF, monomethyl fumarate (MMF), cardiovascular diseases, antioxidant, anti-inflammatory potential, immunomodulatory actions

## Abstract

Dimethyl fumarate (DMF) is a small molecule that has been shown to assert potent in vivo immunoregulatory and anti-inflammatory therapeutic actions. The drug has been approved and is currently in use for treating multiple sclerosis and psoriasis in the USA and Europe. Since inflammatory reactions have been significantly implicated in the etiology and progression of diverse disease states, the pharmacological actions of DMF are presently being explored and generalized to other diseases where inflammation needs to be suppressed and immunoregulation is desirable, either as a monotherapeutic agent or as an adjuvant. In this review, we focus on DMF, and present an overview of its mechanism of action while briefly discussing its pharmacokinetic profile. We further discuss in detail its pharmacological uses and highlight its potential applications in the treatment of cardiovascular diseases. DMF, with its unique combination of anti-inflammatory and vasculoprotective effects, has the potential to be repurposed as a therapeutic agent in patients with atherosclerotic cardiovascular disease. The clinical studies mentioned in this review with respect to the beneficial effects of DMF in atherosclerosis involve observations in patients with multiple sclerosis and psoriasis in small cohorts and for short durations. The findings of these studies need to be assessed in larger prospective clinical trials, ideally with a double-blind randomized study design, investigating the effects on cardiovascular endpoints as well as morbidity and mortality. The long-term impact of DMF therapy on cardiovascular diseases also needs to be confirmed.

## 1. Introduction

Esters derived from fumaric acid have been explored and put to use for therapeutic applications since their introduction for the treatment of psoriasis in 1959 by the German chemist Walter Schweckendiek. The first commercially available fumaric acid ester (FAE) formulation, Fumaderm, was licensed in Germany in 1994 as the first-line choice of systemic therapy for moderate-to-severe plaque psoriasis. Since then, FAEs have become the most frequently prescribed systemic anti-psoriatic medication in Germany [1]. Fumaderm consists of dimethyl fumarate (DMF) as the main prodrug component, which is rapidly hydrolyzed in vivo into the pharmacologically active compound monomethyl fumarate (MMF). In 2013, the United States Food and Drug Administration (USFDA) authorized and approved Tecfidera as the first DMF-based formulation for use in the USA, as the first-line therapy of choice for adult patients with relapsing multiple sclerosis, showcasing a positive safety and powerful efficacy profile [2]. Promising preclinical and clinical data also position DMF as a favorable drug candidate for the treatment of psoriasis in the USA. The European Medicines Agency in 2017 further approved Skilarence—a new oral formulation of DMF—for the treatment of moderate-to-severe plaque psoriasis throughout Europe [1].

DMF has been demonstrated to exhibit potent anti-inflammatory, antioxidative, and immunomodulatory actions in a variety of cell types and tissues [3,4,5]. These effects can be extrapolated to a number of disease states to bring about significant improvements in therapeutic outcomes. Due to its anti-inflammatory, antioxidative, and immunomodulatory properties, the beneficial effects of DMF have been observed in many disease states wherein oxidative stress and/or inflammatory mediators play a central role, such as in amyotrophic lateral sclerosis [6]. Similarly, these beneficial therapeutic effects can be extended to other conditions associated with oxidative stress and/or inflammation, such as migraine, epilepsy, Alzheimer’s disease, Parkinson’s disease, pancreatitis, and nephronophthisis [6,7,8,9]. Its anti-inflammatory potential has generated widespread interest, and it is one of the medications being evaluated for the treatment of COVID-19 infection-induced cytokine storm and acute respiratory distress syndrome [10].

A growing number of preclinical studies have demonstrated the protective therapeutic effect of DMF in atherosclerosis, stroke, myocardial infarction, diabetic cardiovascular complications, and hypertension. The clinical use of DMF over recent decades has provided ample patient-based evidence to establish its safety profile, encouraging the repurposing of DMF for various conditions.

Based on this, the present review primarily focuses on evaluating and expanding the therapeutic applications of DMF for cardiovascular diseases. We analyzed and described the pharmacokinetic profiles of DMF and its major molecular targets, especially with respect to its anti-inflammatory and antioxidative activity. We further comprehensively reviewed the preclinical and clinical evidence supporting its protective effects and applications for the treatment of a variety of cardiovascular diseases.

## 2. Therapeutic Potential of DMF in Cardiovascular Diseases

DMF is Food and Drug Administration (FDA)- and European Medicines Agency (EMA)-approved for the treatment of multiple sclerosis and psoriasis. Currently, various clinical trials are employing DMF as a therapeutic agent to explore its efficacy in treating lymphomas, obstructive sleep apnea (OSA), and adult brain glioblastoma [11]. DMF has been investigated for its therapeutic effects in cutaneous T-cell lymphoma (CTCL) in a phase II clinical trial (NCT02546440) [12]; DMF presented promising results and was found to be well tolerated in CTCL patients [13]. Furthermore, a randomized, placebo-controlled phase II clinical trial was conducted to determine the effectiveness of DMF (NCT02438137) for the treatment of OSA, wherein DMF was found to attenuate the severity of the disease [14]. Suppression of the NF-κB inflammatory pathway was believed to mediate DMF’s therapeutic actions in OSA. DMF has also been investigated in glioblastoma multiforme (NCT02337426), and in rheumatoid arthritis along with methotrexate (NCT00810836); however, the results and conclusions from the trials have not yet been published. Several preclinical studies also support the use of DMF for various other pathological conditions.

**Figure 1:**
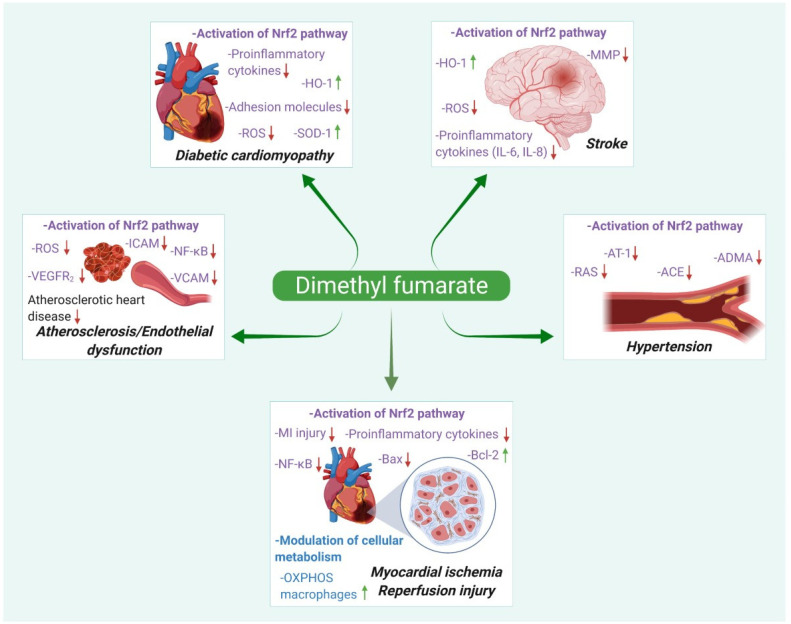
Different pharmacological actions and summarized mechanisms of action of dimethyl fumarate in cardiovascular diseases.

Therefore, DMF may find future applications in the treatment of diseases characterized by inflammation and oxidative stress, which include a wide variety of conditions, such as retinopathy, osteoarthritis, chronic pancreatitis, cancers, inflammatory bowel disease, sepsis, neurodegenerative disorders, and neuromuscular disorders [15,16]. Here, we summarize the beneficial therapeutic effects of DMF, focusing on cardiovascular diseases such as atherosclerosis, stroke, myocardial infarction, diabetic cardiomyopathy, and hypertension. Figure 1 depicts the pharmacological actions and possible therapeutic indications of DMF in various cardiac pathologies.

### 2.1. Atherosclerosis

Atherosclerosis is the principal cause of cardiovascular-related deaths occurring worldwide. Cholesterol-rich fatty plaques are deposited in arteries as a result of multiple interconnected mechanisms resulting from inflammatory reactions, free-radical-species-mediated oxidative stress, lipid dysregulation, and epigenetic disorder [17,18,19]. As the atheromatous plaques progress towards advanced stages, they become highly vulnerable to rupture, leading to severe cardiovascular events such as ischemic stroke and myocardial infarction [20]. The anti-inflammatory actions of DMF have been suggested to provide potential benefits in impeding the development of atherosclerosis.

Luo et al. investigated these benefits in vivo in a diabetic model of atherosclerosis. Hyperglycemia-induced oxidative stress has been shown to aid in vascular endothelial injury, and an increased inflammatory response in atherosclerosis is associated with diabetes mellitus [21]. With the use of DMF at an oral dose of 25 mg/kg in a model of STZ-induced diabetic ApoE-deficient mice, they observed a concurrent increase in the expression of antioxidant Nrf2, reduction in aortic oxidation, and substantial improvements in endothelial functions of the thoracic aorta. Nrf2 regulates oxidative stress in vivo, and the activation of the Nrf2/ARE antioxidative pathway reduces the production of ROS, diminishes the inflammatory responses, protects endothelial cells from oxidative damage, and inhibits the growth and migration of vascular smooth muscle cells, thereby further reducing the advancement of atherosclerosis.

In another study, DMF treatment at a lower oral dose of 12.5 mg/kg/day significantly decreased high-cholesterol-diet-induced elevation of serum TC, TGs, and LDL cholesterol in hypercholesterolemic rabbits [22]. Dietary DMF supplementation was demonstrated to decrease the aortic levels of malondialdehyde (MDA), thereby indicating reduced lipid peroxidation, and simultaneously improving the expression of antioxidant enzymes such as SOD. These hypolipidemic effects, along with DMF’s anti-inflammatory and antioxidant potential, can therapeutically reduce the occurrence of atherosclerosis.

Clinically, DMF has been observed to confer anti-atherosclerotic effects in patients with multiple sclerosis and psoriasis. DMF treatment results in a marked improvement in the HDL/LDL and HDL/total cholesterol ratios, along with elevated levels of the anti-atherogenic serum adiponectin. The overall reductions in the levels of serum total cholesterol and triglycerides also demonstrate DMF’s therapeutic benefits as an anti-atherosclerotic drug [23,24,25]. A major pathological mechanism preceding atherosclerosis is the development of vascular permeability and the resulting endothelial dysfunction. Cardiovascular risk factors such as dyslipidemia, arterial hypertension, hyperglycemia, and diabetes promote the development of endothelial dysfunction, characterized by an impairment of the endothelium-dependent vasodilation and pro-coagulant/pro-inflammatory endothelial activities [26]. The plaque formation in atherosclerosis results from endothelial inflammation, followed by ROS generation by macrophages. The reactive oxygen species oxidize the circulating LDL, resulting in the formation of foam cells and lipid buildup [27].

DMF targets the endothelial cells and protects against atherosclerosis by improving endothelial function. Oxidative stress in endothelial cells stimulates multiple biological processes involved in vascular inflammation and injury, including the expression and activation of transcription factors (e.g., NF-kB), upregulation of pro-inflammatory markers (cytokines, interleukins) and cellular adhesion molecules (e.g., ICAM, VCAM, PECAM), stimulation of chemokine production (e.g., MCP-1), and the recruitment of pro-inflammatory cells (e.g., monocytes, macrophages) [28]. In vivo studies in balloon-injury-induced animal models of atherosclerosis have demonstrated that DMF reduces neointimal formation and protects against TNF-α-induced endothelial cell apoptosis and dysfunction via Nrf2-NQO1 modulation [29]. DMF treatment was further found to inhibit vascular smooth muscle cell proliferation through p21 induction, leading to G1 cell cycle arrest.

Pharmacological interventions for the correction of endothelial function represent a novel approach for preventing and treating atherosclerosis. However, impairment of endothelial function by pharmacotherapy may accelerate the progression of cardiovascular pathologies, including atherosclerosis, systemic and pulmonary hypertension, or diabetes. Endothelial toxicity is commonly seen with various chemotherapeutic agents, such as trastuzumab, bevacizumab, and doxorubicin [30]. Agents activating the Nrf2 signaling pathway have been tested in various pathologies. Bardoxolone, an orally available semi-synthetic triterpenoid compound, has been found to be an activator of the Nrf2 pathway. Preclinical studies have demonstrated the beneficial therapeutic effects of bardoxolone in different animal models of kidney disease, including the improvement of murine ischemic acute kidney injury, attenuation of renal interstitial inflammation, and protection against fibrosis in a model of chronic kidney disease [31].

A phase III randomized, double-blind, placebo-controlled clinical trial was designed to determine whether bardoxolone methyl could slow down the progression to end-stage renal disease in patients with stage 4 CKD and type 2 diabetes (Bardoxolone Methyl Evaluation in Patients with Chronic Kidney Disease and Type 2 Diabetes Mellitus—BEACON trial). However, the trial had to be prematurely terminated because of safety concerns related to an increase in adverse cardiovascular events, including heart failure, hospitalizations, and deaths in the bardoxolone methyl group. The adverse events were associated with fluid retention, and were attributed to kidney-specific suppression of the endothelin pathway, resulting in sodium and volume retention [32]. The lessons gained from the trial and the identification of various risk factors are being applied in ongoing clinical trials for bardoxolone methyl in patients with Alport syndrome and chronic kidney disease (Tsubaki study and Ayame study) [33].

In addition to the benefits for endothelial function in animal models, DMF has been found to improve endothelial function in various in vitro studies as well. Upon treatment with DMF, the adhesion of monocytes to activated human umbilical vein endothelial cells (HUVECs) was significantly reduced. This effect can be attributed to the downregulated expression of genes implicated in adhesion, such as the vascular cell adhesion molecule-1 (VCAM-1), intercellular cell adhesion molecule-1 (ICAM-1), and E-selectin [34,35,36]. Loewe et al. further established that DMF impaired the nuclear translocation of NF-κB, resulting in the inhibition of TNF-α-induced tissue factor protein expression in endothelial cells [37]. DMF also exerts antiangiogenic effects via the downregulation of VEGFR-2 expression in human endothelial cells [38,39]. Additional investigations have demonstrated its ability to suppress various TNF-α-induced pro-inflammatory and pro-atherogenic cytokines/chemokines (i.e., MCP-1, CCL-5, PDGF-BB, GM-CSF, and IL-6) in human endothelial cells [40]. These studies suggest that DMF might play a beneficial role in patients with atherosclerosis and endothelial dysfunction.

### 2.2. Stroke

Stroke, also known as cerebral ischemia, is one of the leading causes of death globally, and on the basis of its developmental etiology is primarily derived from hemorrhagic complications or ischemia, with the latter being more common [41]. Ischemic stroke is caused by the interruption of the blood supply to the brain, resulting either from blood vessel rupture or its blockage by a thrombus or embolus. Inflammation and oxidative stress are essential hallmarks of the pathogenesis of ischemic stroke, causing neuronal malfunction and cell death.

The neuroprotective effects of DMF in vivo have been studied in detail in cerebral artery occlusion and bilateral common carotid artery occlusion models of cerebral ischemia, and DMF has been demonstrated to reduce the severity of neuronal damage/cell death upon ischemic insult [42,43,44,45,46]. The primary mechanism of DMF could be attributed to its ability to activate the nuclear factor erythroid 2-related factor 2 (Nrf2) pathway—an important component to activate and sustain antioxidant defense. This upregulates several Nrf2 target genes (i.e., *Gclm, Nqo1*, *and Ho-1*) in the brain [47,48]. DMF exerts antioxidant potential and inhibits the inflammatory responses in the ischemic area by decreasing the levels of pro-inflammatory cytokines [42,49]. These preclinical findings are promising, and show that DMF treatment improves neuronal survival upon ischemic stress while reducing neurological deficits and infarct volume.

Lin et al. observed that DMF not only acts as an antioxidant, but also possesses immunomodulatory properties. This suppresses the infiltration of neutrophils and T cells, as well as reduce the numbers of activated microglia/macrophages in the infarct region. Concurrently, pro-inflammatory cytokine levels were greatly diminished [42]. Lin-Holderer et al. further studied the effects of DMF on murine hippocampal slice cultures and neuronal cell lines (HT-22 and SH-SY5Y) [47]. The ischemic damage in the slice cultures and cell lines was stimulated by depriving them of oxygen and glucose. DMF treatment (30–100 μM) activated the Nrf2 pathway and reduced cell death in hippocampal cultures upon reoxygenation, and promoted neuronal survival upon exposure to ischemic stress.

DMF has also been shown to be beneficial in other acute cerebrovascular conditions, such as hemorrhagic stroke and subarachnoid hemorrhage [50,51]. Zhao et al. provided therapeutic evidence related to systemic DMF therapy. In the experimental model, DMF (15 mg/kg) was initially administered intraperitoneally two hours after ICH, followed by oral administration twice a day on days 1–3. Treatment with DMF improved hematoma resolution, attenuated functional deficit and brain edema, and enhanced neurological recovery within a 24 h therapeutic window in a rodent model of acute intracerebral hemorrhage (ICH)—a devastating form of stroke [51]. Additional experiments were conducted in wild-type and Nrf2-knockout mice. In wild-type mice, DMF upregulated the expression of Nrf2 as well as its downstream targets, including heme oxygenase-1 (HO-1) and catalase. In addition, the expression of the genes haptoglobin (a hemoglobin-detoxifying protein) and CD163 (the receptor for the hemoglobin–haptoglobin complex), which plays a potential role in ICH, was also induced by DMF. Upregulation of these proteins was attenuated in Nrf2-knockout mice. DMF mediated the improvement in hematoma resolution by stimulating the phagocytotic activity of microglial cells.

The neuroprotective effect of DMF (100 mg/kg, i.p.) was also demonstrated by Iniaghe et al. in a murine model of ICH [52]. Intrastriatal injections of bacterial collagenase and autologous blood were used to develop models of intracerebral hemorrhage. DMF was found to attenuate functional deficits and brain edema. The reduction in brain edema could be attributed to the preservation of blood–brain barrier integrity by DMF. Furthermore, treatment with DMF reduced ICAM-1 levels and increased the levels of casein kinase 2.

Ischemic stroke and reperfusion injury are often associated with disruption of the integrity of the blood–brain barrier. Reperfusion injury includes oxidative stress, platelet activation, inflammatory responses, and neuronal apoptosis [53]. Therefore, DMF is a high-potential therapeutic candidate in ischemia–reperfusion injury, due to its anti-inflammatory and antioxidant potential. Pretreatment with DMF (15 mg/kg) has been examined to show its capability of maintaining the integrity of the blood–brain barrier by preventing the disruption of interendothelial tight junctions, decreased matrix metalloproteinase activity in brain tissue, and diminished cerebral edema formation [49]. DMF strongly enhanced the expression of Nrf2 and related target genes, such as HO-1, in endothelial cells and astrocytes. Treatment with DMF also resulted in attenuation of pro-inflammatory factors and inhibited leukocyte transmigration. The protective effect of DMF against ischemia–reperfusion brain injury in Nrf2-gene-deficient mice was evaluated by Yao et al. The infarct volume and cerebral edema in DMF (45 mg/kg)-treated Nrf2-gene-deficient mice were comparable to those of vehicle-treated Nrf2-gene-deficient mice. The levels of HO-1 mRNA were also not changed with DMF treatment. These results suggest that the Nrf2 signaling pathway is crucial for DMF to exert neuroprotective effects in cerebral ischemia–reperfusion injury [43].

Neuronal death results from a deficient supply of oxygen and concomitant oxidative stress and inflammation in the ischemic core. A therapeutic agent that augments cellular resistance to oxidative stress and exerts immunomodulatory responses can provide enhanced protection after ischemic stroke. DMF, with its antioxidative and immunomodulatory actions, could be beneficial as a potential neuroprotective agent in the treatment of stroke. Table 1 lists the ongoing clinical trials for DMF in the treatment of various forms of stroke.

### 2.3. Myocardial Infarction/Ischemia—Reperfusion Injury

Ischemic injury in a tissue leads to a deficient supply of oxygen, glucose, and other nutrients required for cellular metabolism, ultimately leading to damage to the stressed tissue. Replenishing the perfusion to the tissue by clearing the obstructed artery is an effective treatment for acute myocardial infarction (MI); however, reperfusion may itself give rise to myocardial injury. During ischemia, the degradation of ATP produces hypoxanthine, and upon reperfusion, an influx of molecular oxygen degrades it to uric acid, thereby liberating the highly reactive superoxide anion (O_2_^−^). Superoxide free radicals are subsequently converted to hydrogen peroxide (H_2_O_2_) and the hydroxyl radical (OH^•^). Such reactive species lead to the peroxidation of the cell membrane associated with lipid structures, disrupting the cells’ permeability and functionality, and eventually leading to cell death. During the ischemia-associated reperfusion injury, ROS act upon the endothelial cells and activate the NF-κB pathway. Once activated, the endothelial cell produces E-selectin, VCAM-1, ICAM-1, plasminogen activator inhibitor-1 (PAi-1), tissue factor, and interleukin-8 (IL-8), leading to inflammatory damage in the tissue region surrounding the infarct [54].

Reperfusion injury involves both oxidative stress and inflammation, and DMF could be a powerful potential therapeutic candidate to prevent myocardial tissue damage. Conventional drugs—such as β-blockers, ACE inhibitors, and statins—in combination with timely reperfusion improve the prognosis in patients with acute MI [55]. Preclinical studies in various animal models have shown an equivalent protective effect of DMF in reperfusion injury after stroke. The administration of DMF reduced oxidative stress, inflammation, and brain edema by preserving the blood–brain barrier’s integrity. A reduction in infarct size and improved motor and sensory function were also observed with DMF treatment.

DMF exerts its cardioprotective effects by activating the Nrf2 antioxidant pathway [56]. Kuang et al. conducted in vitro investigations to assess the beneficial role of DMF in myocardial ischemic reperfusion injury. The treatment with DMF improved cellular viability and enhanced the expression of Nrf2-regulated genes [57]. This was accompanied by suppression of apoptotic cell death markers. The production of reactive oxygen species also decreased with DMF administration. Pretreatment of rat H9c2 cardiomyoblast cells with DMF (20 µM) attenuated cellular damage and improved viability. The addition of DMF significantly decreased the levels of Bax and cleaved caspase-3, while the Bcl-2 expression was upregulated, indicating its potential antiapoptotic effect. A significant decrease was seen in the number of early apoptotic cells, whereas that of living cells was notably increased. In addition to the suppression of ROS production, DMF increased the expression levels of the free radical scavengers, HO-1, and NQO1. DMF treatment markedly elevated the levels of p-Akt/Akt and Nrf2, indicating that DMF activity could be attributed to activation of the Akt/Nrf2 pathway.

Zhao et al. further assessed the protective role of DMF in vitro using H9c2 cardiomyocytes. In the study, an oxygen–glucose deprivation/reperfusion (OGD/R) model was used to simulate myocardial ischemia/reperfusion injury. The results demonstrated the protective capacity of DMF (20 µM) through its inhibition of pro-inflammatory cytokines and chemokines (e.g., IL-6, IL-8, and MCP-1), reduced generation of ROS, and downregulation of adhesion molecules (e.g., TF, ICAM-1) [58]. The antioxidant effect was mediated through the inhibition of NOX4-mediated ROS production. DMF mediated the downregulation of adhesion molecules through the suppression of the Egr-1 signaling pathway. Early growth response-1 (Egr-1) is a transcriptional factor upregulated in response to ischemia and reperfusion, and initiates the expression of chemokines, adhesion molecules, and coagulant factors. Furthermore, the investigations in rat heart endothelial cells established the inhibitory effect of DMF on the nuclear translocation of NF-κB [59].

Additionally, preclinical studies by Meili-Butz et al. demonstrated the capacity of DMF to reduce the myocardial infarct size in a rodent model of myocardial infarction, through attenuation of the NF-κB pathway [59]. The infarct size in rats that had received DMF (10 mg/kg) was significantly smaller in comparison to vehicle-treated control rats, suggesting the anti-necrotic effect of DMF. Valen et al. described the detrimental role of cardiac NF-κB in reperfusion and infarction [60]. The cardioprotection via NF-κB inhibition limits myocardial infarct size during ischemia–reperfusion injury. In another study, the modulation of cellular metabolism by DMF was found to protect against MI-induced cardiac injury [61]. Adult male C57BL/6J mice were treated with DMF (10 mg/kg, 3–7 days) after MI. DMF treatment was found to attenuate LV infarct wall thinning following MI, and to decrease LV dilation and pulmonary congestion. Improved LV infarct collagen deposition, myofibroblast activation, and angiogenesis were also seen with DMF treatment. DMF reduced inflammatory markers and enhanced Nrf2 expression. DMF was also found to affect macrophage metabolism by promoting mitochondrial oxidative phosphorylation in macrophages and augmenting anti-inflammatory activities.

Sun et al. have demonstrated the inhibitory effect of DMF on dendritic cell maturation and cytokine secretion. Dendritic cells play an important part in atherogenesis—particularly in plaque rupture, which contributes to myocardial infarction [62]. DMF also inhibited the expression of the costimulatory molecule CD86, the chemokine receptor CCR7, and the C-X-C motif chemokine receptor 4 (CXCR4). DMF has also exhibited a protective role in ischemic injury of organs such as the lungs, liver, and kidneys [63,64,65]. Timely reperfusion and the inhibition of I/R injury are crucial for the management of myocardial ischemia. Taking into consideration the protective effects of DMF in myocardial I/R injury, DMF-mediated therapy could be of immense clinical significance.

### 2.4. Cardiovascular Complications Associated with Diabetes

Diabetes is a worldwide progressive metabolic disorder, and the associated complications can have severe effects on the quality and expectancy of life. Among the various complications, diabetic cardiomyopathy significantly contributes to disease mortality. ROS-mediated oxidative stress is considered to be one of the major causes of the pathogenesis of this disease, leading to cardiomyocyte damage [66]. Studies have shown that the Nrf2/ARE pathway protects against oxidative cardiac cell injury as well as diabetic complications [67]. DMF, an Nrf2 activator with antioxidant and anti-inflammatory potential, can be considered as a novel therapeutic agent for the treatment of diabetic cardiomyopathy.

DMF has exhibited significant beneficial effects in various animal models of diabetic cardiomyopathy and associated vascular complications. Hu et al. investigated the effect of DMF on the development of diabetic cardiomyopathy in animal models. Type 1 diabetes in mice was established using multiple low doses of streptozotocin. The diabetic mice were then treated with DMF (10 mg/kg, i.p.) for 3 months [68]. Administration of DMF effectively attenuated inflammation and oxidative stress in diabetic hearts. DMF treatment decreased the levels of pro-inflammatory cytokines and adhesion molecules. DMF also inhibited myocardial dysfunction and cardiac fibrosis. In addition, activation of Nrf2 and its downstream antioxidants (HO-1, SOD1, and catalase) was observed with DMF treatment, which resulted in the cardioprotective response. These findings suggest that DMF could prevent diabetes-induced myocardial tissue injury via activation of Nrf2 function.

DMF was also found to be beneficial in diabetes-associated vascular complications.

Amin et al. tested the effect of oral DMF treatment (25 mg/kg) on diabetic vascular dysfunction. Treatment with DMF reduced aortic levels of ROS and NF-κB-65, and restored aortic GSH, SOD, and Nrf2. This was accompanied by a reduced expression of inflammatory mediators (e.g., IL-1β, TNF-α, TGF-β) in the vessel walls. DMF increased eNOS mRNA and protein levels and increased bioavailable NO in diabetic aortas. Fibrous tissue proliferation in aortic tunica media was also alleviated. The vasculoprotective effect of DMF on diabetic aortas can be attributed to attenuation of the ROS-TXNIP-NLRP3 inflammasome pathway. Inhibition of TXNIP suppresses the activation of NF-κB and its downstream inflammatory genes [69].

Lu et al. reported the restoration of vascular BK channel function through activation of Nrf2 by DMF. High-fat diet (HFD)-induced obese/diabetic mice were used for the study. Increased oxidative stress in the diabetic vessels impairs BK channel function because of the NF-κB-mediated protein degradation of its beta-1 (BK-β1) subunits. BK channels are critical for vasodilation, and DMF treatment (25 mg/kg) preserved BK-β1 expression and restored BK channel function in the coronary arteries [70]. Hence, Nrf2 activation by DMF can inhibit cardiovascular diseases related to diabetes, as well as its vascular complications.

Vascular calcification is highly prevalent in patients with diabetes, metabolic syndrome, atherosclerosis, hypertension, and chronic kidney disease. [71]. Prophylactic oral DMF treatment (25 and 50 mg/kg) prevented vitamin-D3-induced calcification in mice, in a dose-dependent manner. Studies of ex vivo ring cultures from rat common carotid artery and murine thoracic aorta tissues showed decreased vascular calcification with DMF treatment [72].

In addition to diabetic cardiomyopathy, Nrf2 plays a protective role in diabetic nephropathy and diabetic retinopathy [31]. Lone et al. demonstrated the renoprotective potential of dimethyl fumarate in streptozotocin-induced diabetic nephropathy in Wistar rats [73]. DMF was also reported to accelerate wound healing under diabetic conditions, which is a common complication among patients with diabetes mellitus (DM) [74]. There is a consistent decrease in oxidative stress and inflammation with DMF treatment. These studies are promising, and suggest that DMF could be broadly used in various diabetic complications.

### 2.5. Hypertension

Inflammation, oxidative stress, and vascular dysfunction play a vital role in the pathogenesis of hypertension [75,76]. Farooqui et al. reported that Nrf2 inhibition aggravated oxidative stress and inflammation, thereby heavily contributing to the development of hypertension [77]. Hsu et al. investigated the effects of maternal DMF therapy (50 mg/kg/day) in adult rat offspring against dexamethasone and high-fat-diet-induced programmed hypertension. Prenatal dexamethasone (DEX) exposure leads to hypertension in adult offspring, which is aggravated by a postnatal high-fat (HF) diet. Maternal DMF therapy exhibited protective effects in the male offspring against the development of hypertension [78]. Treatment with DMF reduced renal mRNA expression of RAS components (i.e., renin angiotensinogen, angiotensin converting enzyme-1, and angiotensin II type1 receptor), resulting in the downregulation of the renin–angiotensin system, and leading to the protective effects of DMF.

DMF also promoted Nrf2 mRNA expression. Simultaneously, a significant decrease in ADMA plasma levels (asymmetric dimethylarginine), which are associated with adverse effects on cardiovascular health, was also observed. The reduction in ADMA levels restored the NO–ROS balance and increased NO bioavailability to prevent DEX+HF-induced hypertension. Their findings also suggested that DMF stimulated autophagy and reduced oxidative stress. An increase in the mRNA expression of autophagy-related genes—such as UNC-51-like kinase-1 (Ulk1) and autophagy-related gene 5 (Atg5)—was observed in the kidneys of the offspring. In a similar model of DEX+HF-induced hypertension, Lin et al. reported that early postnatal treatment with DMF (50 mg/kg/day for 3 weeks after weaning) protected against hypertension. DMF significantly reversed DEX+HF-induced hypertension, increased levels of Nrf2, and protected against oxidative stress [79]. Further analysis also showed that DMF decreased the plasma ADMA and SDMA levels, and increased mRNA expression of the autophagy-related genes Ppargc1a, Ulk1, and Atg5 in male offspring’s kidneys. Thus, DMF may be effective in preventing hypertension in children exposed to antenatal corticosteroids and high postnatal intake of fats.

In another study, Grzegorzewska et al. reported the beneficial effects of DMF by employing an experimental model of pulmonary arterial hypertension and lung fibrosis. DMF was tested in a hypoxia model and a hypoxia/SU5416 mouse model. In the hypoxia/SU5416 mouse model, mice were exposed to hypoxia for 3 weeks with weekly injections of 20 mg/kg SU5416 (vascular endothelial growth factor receptor antagonist, Sugen 5416). DMF treatment was found to be effective in reversing the hypertension-related hemodynamic changes and decreasing inflammation, oxidative injury, and fibrosis. DMF (90 mg/kg, i.p.) significantly improved pathological hemodynamics in hypoxic mice. It was found to prevent and reverse elevated right ventricular systolic pressure and RV hypertrophy in the murine model. DMF inhibited the NF-κB and STAT pathways and reduced expression of IL-6 in vitro, further confirming its antioxidative and anti-inflammatory actions [80]. The beneficial effects of DMF in hypertension are promising; however, further preclinical and clinical studies are necessary to establish its clinical potential.

### 2.6. Other Cardiac Pathologies

DMF has been tested following a prophylactic protocol in animal models of cardiac hypertrophy [81]. In a murine model of isoproterenol (ISO)-induced cardiac hypertrophy, DMF (25 mg/kg, orally) was found to decrease heart rate and systolic and diastolic blood pressure. A significant improvement was also observed in the ECG pattern and cardiac rhythm. Cardiac troponin-I, creatine kinase-MB, and atrial natriuretic peptide levels decreased with DMF treatment. Pretreatment with DMF also reduced oxidative stress and suppressed NF-κB and other pro-inflammatory cytokines.

DMF exerts its cardioprotective effects by disrupting the TLR-mediated inflammatory process and the MAPK pathway. DMF decreased the levels of the TLR adaptor protein MyD88, followed by reductions in NF-κB, TNF-α, IL-6, and p-ERK1/2 levels. However, additional preclinical studies are required in order to develop DMF therapies for clinical testing and implementation. Another common cardiac pathology is heart failure. Since inflammation and oxidative stress play important roles in the development of heart failure [82], future studies are essential to understand the potential therapeutic benefits of DMF.

### 2.7. Aneurysm

Chronic inflammation in arterial walls because of hemodynamic stress leads to the formation of intracranial aneurysm. Pascale et al. examined the protective role of DMF in preventing the formation and rupture of intracranial aneurysm in vitro and in vivo. DMF (20, 50, 75, and 100 µM) was evaluated in vitro using TNF-α-treated vascular smooth muscle cells, and in vivo using a murine-elastase-induced model of aneurysm [83]. The mice were administered DMF (100 mg/kg, orally) for two weeks. DMF therapy was shown to protect the cells by activating the Nrf2 pathway and decreasing the production of pro-inflammatory cytokines and chemokines.

At higher doses, DMF increases apoptosis and protects the cells from inflammatory necrotic cell death by suppressing the pro-proliferative action of TNF-α. Thus, experimental models present preclinical data suggesting that DMF-treatment-mediated upregulation of Nrf2 significantly reduces the aneurysm formation and rupture. These studies suggest that DMF, with its inhibitory effects on oxidative stress, inflammation, and fibrosis in the cerebrovascular region, can play a potential role as a therapeutic agent for patients at risk of the formation and rupture of intracranial aneurysms; however, more preclinical studies are required to establish its clinical role.

## 3. The Mechanisms of Action of DMF

**Figure 2:**
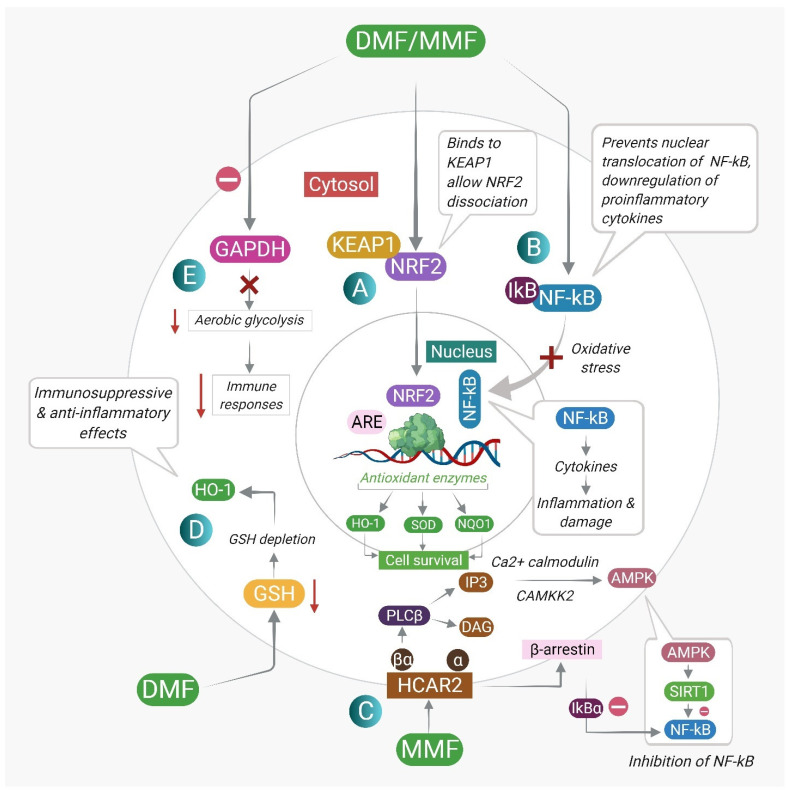
The different mechanisms of action of DMF and its downstream targets: (**A**) DMF reacts with cysteine residues of Keap1, leading to dissociation of Nrf2 from Keap1 and nuclear translocation of Nrf2. Nrf2 binds to the antioxidant response element (ARE) and drives the expression of antioxidant target genes, promoting cell survival. (**B**) DMF prevents the nuclear translocation of NF-κB. Inhibition of NF-κB leads to inhibition of pro-inflammatory cytokine production and inhibition of inflammation and cellular damage. (**C**) MMF, a potent agonist of the HCAR2 receptor, interacts with β-arrestin and inhibits NF-κB. Activation of HCAR2 upon MMF binding also leads to the activation of AMP-activated protein kinase (AMPK) by calcium/calmodulin-dependent protein kinase 2 (CaMKK2), which further leads to the activation of the protein deacetylase sirtuin-1 (SIRT1), resulting in inhibition of NF-κB signaling. (**D**) DMF induces a transient GSH depletion by forming DMF–GSH conjugates, leading to HO-1 production. The initial depletion of GSH is followed by a rebound increase in GSH concentrations, which facilitates GSH-dependent detoxification pathways. (**E**) DMF inhibits aerobic glycolysis in immune cells, inhibiting the activation of immune cells and promoting the development of anti-inflammatory immune cell subsets.

The therapeutic effects of DMF are exerted through the modulation of cellular proteins and their signaling pathways. These include the nuclear factor erythroid 2-related factor 2 (Nrf2) antioxidant pathway, the nuclear factor kappa B (NF-κB) pathway, GSH modulation, inhibition of aerobic glycolysis, and agonism of hydroxycarboxylic acid receptor 2 (G-protein-coupled receptor 109A). Figure 2 describes the different DMF-mediated molecular mechanisms and downstream targets. Table 2 summarizes the major mechanisms of action responsible for the therapeutic effects of DMF. These targets, along with their downstream interactions, are discussed in detail in this section.

### 3.1. Nrf2 Antioxidant Pathway Activation

DMF has been shown to be a potent activator of Nrf2 and its downstream targets. The transcription factor Nrf2 is an endogenous cellular defense mechanism against oxidative stress, and regulates the cellular redox homeostasis [87]. The role of Nrf2 has been implicated in metabolic and cardiovascular diseases, neurodegenerative conditions, and cancer, as well as respiratory disorders [12] The transcription factor Nrf2 can be pharmacologically targeted, rendering it clinically significant for drug development and repurposing. It is regulated by the Keap1-Nrf2 pathway. Under normal conditions, Nrf2 is maintained in the cellular cytoplasm in its inactive form, bound to the repressor molecule Kelch-like ECH-associated protein 1 (Keap1). Keap1 serves as a substrate adaptor protein for Cullin3/Rbx1 ubiquitin ligase, facilitating Nrf2 ubiquitination. Keap1 sequesters and polyubiquitinates Nrf2 in the cytosol, leading to its constitutive degradation. Under physiological conditions, Nrf2 has a short half-life of only 20 min [88]. Under stressful conditions, oxidation of cysteine residues in Keap1 disrupts the Keap1 ubiquitination system, promoting the dissociation of Nrf2 from its complex in the cytoplasm, followed by its translocation into the nucleus [89].

Keap1 is composed of a three-domain architecture, and consists of an N-terminal BTB (broad complex, tramtrack, and bric-a-brac) domain, an intervening region (IVR) or BACK domain, and a C-terminal Kelch repeat domain. The Kelch domain binds to the Neh2 domain of Nrf2 at two amino acid sequences: DLG and ETGE. Nrf2 activators are mostly electrophilic molecules that covalently modify cysteine residues present in the thiol rich Keap1 protein via oxidation or alkylation.

Two different conformations are adopted by the Nrf2/Keap1 complex, sequentially alternating in a cycle in basal conditions. The open conformation is formed by binding of newly synthesized Nrf2, through its high-affinity ETGE motif, to one Keap1 molecule of the Keap1 dimer. This prevents Nrf2 ubiquitination and protects Nrf2 from proteasomal degradation. After a period in the open conformation, the cycle moves to form the closed conformation [90]. In the next step, the low-affinity DLG motif of Nrf2 binds to the second member of the Keap1 dimer. Thus, the complex adopts a closed conformational state, which predisposes Nrf2 to polyubiquitination and proteasomal degradation. The regenerated free Keap1 dimer is then able to bind to newly synthesized Nrf2, and the cycle begins again. Nrf2 activators block the Nrf2/Keap1 complex in the closed conformation through modifications of cysteines in Keap1. Inhibition of the proteasomal degradation of Nrf2 blocks the cycle, leading to the accumulation of the Keap1–Nrf2 complex in the closed conformation, and free Keap1 is not regenerated at a sufficient rate. Hence, the mechanism of Keap1 inhibition by Nrf2 activators includes sequestration in complexes with Nrf2 that cannot be ubiquitinated. Therefore, transactivation of Nrf2 target genes is achieved by de novo synthesized Nrf2, which escapes Keap1-mediated ubiquitination and degradation, and translocates to the nucleus [91].

Nrf2 activators also disrupt the interaction between Keap1 and Cullin3/Rbx1, resulting in clogging of Keap1 in an Nrf2-bound conformation, and newly synthesized Nrf2 escapes ubiquitination [92]. Several (non-FAE-based) Nrf2 inducers have been developed and tested in clinical trials in recent years (e.g., ursodiol, sulforaphane, etc.), and have met with considerable success with regard to potential for clinical development [92]. After its translocation into the nucleus, Nrf2 interacts with antioxidant response elements of target genes and induces transcriptional activation of NAD(P)H quinone oxidoreductase 1 (NQO1), heme oxygenase-1 (HO-1), glutathione-S-transferase, and direct antioxidants (e.g., superoxide dismutase (SOD), catalase, glutathione reductase and glutathione peroxidase) [93]. Many of the Nrf2-regulated enzymes are essential in the pathogenesis of cardiovascular diseases [94,95].

Recent studies have shown that the mitogen-activated protein kinase (MAPK), phosphatidylinositol-3 kinase (PI3K/Akt) signaling, and multiple epigenetic factors are conducive to the activation of the Nrf2 signaling pathway. [96,97,98]. Nrf2 is able to amplify a wide range of cellular defense mechanisms, enhancing the overall capacity of cells to detoxify potentially harmful substances. It can be targeted for alleviating oxidative stress and associated diseases. The human Keap1 protein is a cysteine-rich protein that contains 27 cysteine residues, which constitute 4.33% of all amino acids, whereas the average content of cysteines in human proteins is 2.26%, making it a strong electrophile trap. Keap1 cysteines are highly reactive due to the surrounding positively charged, basic amino acids [91].

DMF, due to its electrophilic moiety, causes conformational changes in the Keap1 protein by succination of its cysteine residues—particularly cysteine 151, located in the BTB domain. DMF spontaneously reacts with cysteine (SH) residues in proteins via a Michael addition reaction to form S-(2-succinyl) cysteine (2SC), disrupting its interaction with Nrf2, and resulting in stabilization and accumulation of nuclear Nrf2 [99,100]. Conclusively, DMF promotes the translocation of Nrf2 into the nucleus, and upregulates the expression of antioxidant genes [101]. All of the proteins or metabolites are not equally sensitive to succination by DMF. Succination by DMF is specific to particularly “sensitive” cysteine residues. DMF mostly reacts with low pKa thiols, and their reactivity is also affected by their accessibility within the protein. Fumarate concentration may be vital for accessing the cysteine residues and subsequent succination [11].

### 3.2. NF-κβ Pathway Inhibition

NF-κB is another intrinsic transcription factor regulated by DMF. NF-κB regulates the coordinated expression of a diverse group of genes that include cytokines and adhesion molecules implicated in the pathophysiology of myocardial ischemia–reperfusion injury, ischemic preconditioning, apoptosis, atherosclerosis, unstable coronary syndromes, and heart failure [60]. The NF-κB family consists of the members p50, p52, p65 (RelA), c-Rel, and RelB, which form various homo- and heterodimers. Amongst these, the most common form is the active heterodimer of p50 or p52/RelA. The biological activity, the nucleocytoplasmic distribution, and the DNA binding activity of these proteins are, in turn, controlled by a family of inhibitory proteins, termed IκB, to which NF-κB proteins are bound under unstimulated conditions. Under normal conditions, NF-κB is inactive, and remains as a pair of dimers complexed with IκB in the cellular cytoplasm. During oxidative stress, the IκB kinase (IKK) complex comprising IKKα and IKKβ mediates NF-κB activation by phosphorylating the inhibitor IκB. The phosphorylated IκB is then ubiquitinated and degraded by the proteasome machinery, resulting in the release of NF-κB, which translocates to the nucleus and stimulates gene transcription at the target sites [102].

The NF-κB pathway is activated mostly by the stimulation of pro-inflammatory receptors, such as the TNF receptor superfamily, the toll-like receptor family (TLRs), and cytokine receptors [103]. The TLR-mediated NF-κB activation and subsequent pro-inflammatory cytokine production involve both myeloid differentiation factor 88 (MyD88)-dependent and MyD88-independent pathways. DMF has been shown to suppress extracellular signal-regulated kinases 1 and 2 (ERK1/2), thereby negatively regulating its downstream target—mitogen stress-activated kinase 1 (MSK1). The activity of the transcription factor p65 subunit, which is associated with the formation of NF-κB heterodimers, is hindered via MSK1-mediated suppression, resulting in decreased p65 phosphorylation (at serine 276). DMF activity was further shown to inhibit the nuclear translocation of p65 [104]. TLRs have been found to play an important role in the inflammatory process in cardiovascular diseases [105].

McGuire et al. demonstrated DMF-mediated inhibition of NF-κB and MAPK/ERK1/2 activation in response to TLR agonists [106]. This shows that DMF might interact with TLR-mediated inflammatory processes, the MAPK pathway, and the resultant loss of pro-inflammatory cytokine production. The interaction between the NF-κB signaling and Nrf2 pathways has also been extensively studied [107,108]. The activation of the Nrf2 pathway has been found to attenuate the NF-κB-mediated inflammatory response [109]. NF-κB p65 has been found to negatively regulate the Nrf2 antioxidant response element signaling pathway by competing with Nrf2 for binding to the transcriptional coactivator CREB-binding protein [110]. However, Gillard et al. demonstrated the inhibitory effect of DMF on NF-κB and NF-κB-driven downstream production of pro-inflammatory cytokines, as well as translocation of NF-κB to the nucleus independent of the Nrf2 pathway [111]. Inhibition of NF-κB by DMF leads to a decrease in the production of pro-inflammatory cytokines, distorts the function of antigen-presenting cells, and shifts the cytokine production pattern from type 1 and type 17 (Th1/Th17) to the anti-inflammatory type 2 subset (Th2) [112].

### 3.3. HCAR2 Activation

Numerous studies have implicated MMF—the active metabolite of DMF—as being an agonist of the G-protein-coupled receptor, HCAR2 (hydroxycarboxylic acid receptor 2, GPR109A). This receptor is expressed in adipocytes, neutrophils, monocytes, dendritic cells, macrophages, epidermal Langerhans cells, and microglial cells; however, lymphocytes lack the receptor. The receptor mediates the lipid-modifying and anti-atherosclerotic effects of the drug nicotinic acid [113]. HCAR2 receptor activation and its downstream targets induce a strong anti-inflammatory response, and reduce neutrophil infiltration, adhesion, and chemotaxis. G protein αi and Gβ subunits are involved in HCAR2 signaling pathways. Upon dissociation, Gβ subunits activate protein kinase C and ERK1/2. ERK1/2 are implicated in the expression of ATP-binding cassette transporter A1, which mediates cholesterol efflux. HCAR2 receptors also bind to β-arrestin, which can interact with IκB, and prevent NF-κB activation [114]. DMF modulates the HCAR2 receptor signaling independently from the Nrf2 pathway [115]. 

In a study by Chen et al, the DMF treatment regimen in the experimental model of autoimmune encephalomyelitis reduced neurological deficit and other pathological features in WT mice; however, these effects were not observed in HCAR2-deficient mice, showing the significant effects of DMF mediated through the HCAR2 signaling pathway [84]. Using the same HCAR2-mediated pathway, MMF has been shown to activate the microglial AMPK/SIRT1 signaling pathway. Activation of HCAR2 upon MMF binding leads to the activation of phospholipase C, thereby elevating intracellular Ca^2+^ levels. The increase in Ca^2+^ levels results in the activation of AMP-activated protein kinase (AMPK) by calcium/calmodulin-dependent protein kinase 2 (CaMKK2), which further leads to the activation of the protein deacetylase sirtuin-1 (SIRT1). This results in inhibition of NF-κB signaling, thereby reducing the synthesis of pro-inflammatory cytokines [116]. The activation of the HCAR2 receptor has also shown to induce apoptosis via decreasing cyclic AMP. Flushing—a side effect observed with DMF treatment—results from the interaction of MMF with HCAR2 via the release of prostaglandin 2 [117].

### 3.4. Modulation of Glutathione

Glutathione (GSH) serves as an endogenous antioxidant and protects from oxidative damage. DMF interacts with thiol groups and forms conjugates, thereby impacting GSH’s availability and production. DMF maintains a delicate control of the glutathione levels for its antioxidant effects. The initial depletion of GSH induces heme oxygenase 1 (HO-1) production, which downregulates several inflammatory cytokines, contributing to its immunosuppressive and anti-inflammatory responses [118]. Brennan et al. observed that treatment with DMF initially depleted intra- and extracellular GSH levels in human astrocytes, followed by a gradual increase in GSH levels after 10 h, while MMF increased GSH levels after 24 h of treatment [101].

The GSH concentration is maintained by a controlled modulation between its depletion and formation. The activation of the Nrf2 pathway by DMF induces antioxidative enzymes such as superoxide dismutase, glutathione peroxidase, and glutathione reductase, as well as NADPH-regenerating enzymes, which promote GSH formation [115,117]. Hence, the initial depletion of GSH is followed by a rebound increase in GSH concentrations, facilitating GSH-dependent detoxification pathways. The interaction of DMF with GSH also leads to the inhibition of NF-κB and decreased expression of NF-κB target genes that regulate inflammatory cytokines [11].

### 3.5. GAPDH and Glycolysis Modulation

Glycolysis and oxidative phosphorylation are major metabolic pathways that provide energy to the cells. Glycolysis involves a sequence of metabolic reactions for the conversion of glucose to pyruvate to generate energy. Glycolytic metabolism is oxygen-independent, and is less efficient compared to oxidative phosphorylation for ATP synthesis. The main metabolic pathway in pro-inflammatory immune cells is glycolysis, which generates metabolic intermediates that are required for their activation, proliferation, and immune functions [114].

In contrast, differentiation and functions of anti-inflammatory phenotypes are driven by oxidative phosphorylation. DMF succinates the active thiols Cys150 (murine) and Cys152 (human), leading to the inactivation of the glycolytic enzyme glyceraldehyde 3-phosphate dehydrogenase [119]. This downregulates aerobic glycolysis in myeloid and lymphoid cells, mediating anti-inflammatory effects. DMF and MMF inhibit the function and production of T cells (e.g., Th1, Th17) and macrophages (M1), instead promoting the development of anti-inflammatory immune cell subsets of Th2, Treg, and M2 phenotypes [120].

## 4. Pharmacokinetics of DMF

Upon oral administration, DMF is reported to undergo rapid first-pass metabolism where it is bioactivated by the intestinal esterases and the alkaline pH of the small intestine to its biologically active metabolite MMF. DMF thus has a half-life of approximately 12 min, while its metabolite MMF has a half-life of 36 h [121]. DMF is a BCS Class I drug, showing very good water solubility, with high membrane permeability. Phase I pharmacokinetic studies were conducted in 10 healthy subjects receiving DMF and calcium MEF (120 mg plus 95 mg) under fasting conditions. MMF transiently increased to a median maximal plasma concentration of 0.84 mg/L, and disappeared from the plasma with a median elimination half-life of 44 min. DMF could not be detected in the plasma, which could be due to its rapid hydrolysis to MMF. In another study involving three psoriasis patients being treated with the approved FAE mixture, peak MMF concentrations of 1.2–1.8 mg/L were observed after 3.5–4.0 h, and declined with a half-life of 31–71 min, with no detectable levels of DMF in the plasma [122]. Once liberated, MMF does not undergo degradation in the intestine, and is absorbed in the systemic circulation because its ionizable carboxylic acid moiety prevents further hydrolysis [114]. MMF reaches its peak plasma concentrations after 5–6 h, and is subsequently metabolized via the tricarboxylic acid cycle to fumaric acid, water, and carbon dioxide, and is mainly excreted through the pulmonary route via exhalation [121].

Recent studies have further shown that DMF is not completely hydrolyzed to MMF—a significant part of the dose administered also interacts with GSH and forms GSH conjugates, which are further metabolized to mercapturic acid and excreted via the kidneys [118,123]. A small portion of DMF that might escape hydrolysis in the small intestine enters the blood circulation via the portal vein, to be subsequently metabolized into MMF by the esterases present in the circulation. Presumably, the clearance process remains the same in blood and other tissues as well, resulting in the formation of MMF [124]. Patients with renal impairment do not need dose adjustments with DMF, as kidney/urinary excretion plays a minor role in its metabolism [122]. Furthermore, administration with food has been found to interfere with the intestinal absorption and metabolism of DMF, delaying the peak concentration of MMF [125]. Thus, the majority of the DMF administered reaches the liver in its monoester MMF form.

From pharmacokinetic data of Tecfidera GR capsules obtained in subjects with multiple sclerosis, the T_max_ of MMF was found to be between 2 and 2.5 h. Following administration of 240 mg DMF twice a day with food, the median peak (C_max_) was 1.72 mg/L, and the overall area under the curve exposure was 8.02 h.mg/L, in subjects with multiple sclerosis. The apparent volume of distribution varied between 60 L and 90 L after oral administration of 240 mg of DMF. The plasma protein binding of monomethyl fumarate ranges between 27% and 40%. The pharmacokinetics of Tecfidera in the adolescent patients with RRMS aged 13 to 17 years (*n* = 21) were consistent with the findings observed in adult patients (C_max_: 2.00 ± 1.29 mg/L; AUC_0-12hr_: 3.62 ± 1.16 h.mg/L, which corresponds to an overall AUC of 7.24 h.mg/L) [126].

Based on hepatic metabolism studies, further metabolism of MMF in the liver produces methanol and fumaric acid [114]. Additionally, there is no evidence for the interaction of DMF and its metabolite MMF with human cytochrome P450 enzymes and the P-glycoprotein drug transporters [127]. Hence, DMF has very limited drug interactions. However, multiple metabolic processes could lead to variation in its biodistribution profile, thereby impacting its mechanism and therapeutic efficacy. The physiologically active concentrations of DMF are reported between 5.5 and 50 μM, whereas MMF is found to be active at concentrations ranging between 50 and 150 μM [117]. DMF and MMF have been investigated in different preclinical studies for the treatment of new clinical indications.

## 5. Conclusions and Future Perspectives

Despite the extensive research and development of new chemical entities and therapeutic strategies, cardiovascular diseases and their complications remain the leading cause of death and disability worldwide. Drug repurposing, also known as drug repositioning, is a viable therapeutic strategy for hastening drug discovery by identifying new therapeutic indications for already-established drug products. Repurposing of drugs lowers costs, requires shorter time for approval, and provides an opportunity to extend pharmacological tools. Such therapeutic agents have already been tested for dose tolerance and safety in humans, with detailed information being available about their formulations, pharmacology, and potential adverse effects. Drug repurposing has achieved tremendous success with several pharmaceutical agents (e.g., for Viagra, thalidomide, metformin, raloxifene, minoxidil etc.). Oxidative stress and inflammation work together and potentiate one another. Several experimental studies strongly suggest the central role of inflammation in the onset and progression of cardiovascular disorders. Drug repurposing can help to identify safe and effective drugs for cardiovascular diseases. For instance, anti-inflammatory agents such as colchicine and methotrexate are being evaluated in clinical trials for the treatment of cardiovascular diseases.

DMF is a multimodal pharmaceutical agent with immunomodulatory, anti-inflammatory, and antioxidative properties that has achieved remarkable clinical success with psoriasis and relapsing–remitting multiple sclerosis. Thus, the potential applications of DMF therapy are numerous and extremely broad. The adverse effects associated with DMF therapy are relatively mild, and the benefits undeniably outweigh the risks. However, the vast majority of the efficacy data are still preclinical, and further experimental and clinical studies are required for repurposing of DMF for the management of patients with cardiovascular pathologies.

As exemplified in the review, the dose and the routes of administration of DMF vary in different preclinical animal models as well as in vitro investigations. Therefore, further exploration of suitable administration routes, drug dosage and frequency, and treatment duration are needed to find the most optimal and clinically significant treatment paradigms for the different cardiac pathologies. These investigations should be accompanied by the development of DMF-modified release formulations to impart significantly different pharmacokinetic properties for dose reduction, reduced side effects, and further improvements in therapeutic efficacy.

Randomized interventional clinical trials are currently ongoing with DMF as a monotherapeutic agent, or as an adjuvant in patients with acute ischemic stroke and intracerebral hemorrhage, and the results are anticipated with great interest. The results of the ongoing trials will determine whether DMF can be successfully repurposed as a therapeutic option for stroke.

DMF, with its unique combination of anti-inflammatory and vasculoprotective effects, has the potential to be repurposed as a therapeutic agent for patients with atherosclerotic cardiovascular disease. The clinical studies mentioned in this review with respect to the beneficial effects of DMF in atherosclerosis involve observations in patients with multiple sclerosis and psoriasis in small cohorts and for short durations. The findings of these studies will have to be verified in larger prospective clinical trials, ideally with a double-blind randomized study design, investigating the effects on cardiovascular endpoints as well as morbidity and mortality. The long-term impact of DMF therapy on cardiovascular diseases also needs to be assessed. In conclusion, DMF plays a critical role in protecting against CVDs, including atherosclerosis, MI, stroke, hypertension, and diabetic cardiomyopathy, and this may translate into considerable therapeutic promise. The molecular mechanisms of action responsible for its preventive and therapeutic effects have been demonstrated, ascribed to the favorable modulation of molecular pathways identified in numerous studies. Thus, DMF, with its beneficial therapeutic effects and positive safety and efficacy profile, can be extended to various other indications through drug repurposing strategies.

## Figures and Tables

**Table 1:** Clinical trials for indications of dimethyl fumarate in cardiovascular diseases *.

Disease	Official Title	Clinical Trial (Status)	Clinicaltrial.Gov Identifier
Acute Ischemic Stroke	Combination of the immune modulator dimethyl fumarate with intra-arterial treatment in acute ischemic stroke	Phase II (Not yet recruiting)	NCT04891497
Intracerebral Hemorrhage	Dimethyl fumarate for the treatment of intracerebral hemorrhage	Phase II (Not yet recruiting)	NCT04890379
Acute Ischemic Stroke	Combination of the immune modulator dimethyl fumarate with alteplase in acute ischemic stroke	Phase II (Not yet recruiting)	NCT04890366
Acute Ischemic Stroke	Impact of an immune modulator dimethyl fumarate on acute ischemic stroke	Phase II (Not yet recruiting)	NCT04890353
Systemic Sclerosis-Associated Pulmonary Arterial Hypertension	Dimethyl fumarate in pulmonary arterial hypertension (PAH) associated with systemic sclerosis (SSc-PAH): the effect of DMF on clinical disease and biomarkers of oxidative stress	Terminated	NCT02981082

* This list is compiled from (www.clinicaltrial.gov), accessed on 25 March 2022.

**Table 2:** Summary of the main mechanisms of action ascribed to the therapeutic effects of DMF.

Mechanism of Action	Observed Effects	References
Activation of Nrf2	Regulation of cellular antioxidant responses and activation of cytoprotective and anti-inflammatory factors NQO1, HO-1, glutathione-S-transferase, and antioxidants	[47,52]
Inhibition of NF-κB	Downregulation of the pro-inflammatory cytokines; shifts production of T-helper (Th) cells from the Th1/17 subset to the Th2 type	[37,59,69]
Agonism of HCAR2	Inhibits immune cell infiltration, adhesion and chemotaxis, inhibits NF-κB, and reduces production of pro-inflammatory cytokines	[84]
Inhibition of Aerobic Glycolysis	Inflammatory immune cell subsets deprived of aerobic glycolysis; decreased immune responses	[61]
Depletion of GSH	Induces HO-1 and downregulates several inflammatory cytokines	[85,86]

## Data Availability

This is a review and majority of the article referred are cited appropriately in the manuscript.
